# Supplementary material for: MutS functions as a clamp loader by positioning MutL on the DNA during mismatch repair
Source: Nat Commun. 2022 Oct 3;13:5808. doi: 10.1038/s41467-022-33479-3 (PMC9530208; doi:10.1038/s41467-022-33479-3)
Supplement: Supplementary file 1 — Supplementary Information [file 41467_2022_33479_MOESM1_ESM.pdf]

Supplementary Information

MutS Functions as a Clamp Loader by Positioning MutL on the DNA during Mismatch Repair

Xiao-Wen Yang[1,4], Xiao-Peng Han[1,4], Chong Han[1], James London[2], Richard Fishel[2,3*] and Jiaquan Liu[1,*]

[1] State Key Laboratory of Molecular Biology, Shanghai Key Laboratory of Molecular Andrology, CAS Center for Excellence in Molecular Cell Science, Shanghai Institute of Biochemistry and Cell Biology, University of Chinese Academy of Sciences, Chinese Academy of Sciences, 320 Yueyang Road, Shanghai, 200031, China.

[2] Department of Cancer Biology and Genetics, The Ohio State University Wexner Medical Center, Columbus, OH 43210, USA.

[3] The Molecular Carcinogenesis and Chemoprevention Program, The James Comprehensive Cancer Center, Columbus, OH 43210, USA.

[4] These authors contributed equally: Xiao-Wen Yang, Xiao-Peng Han

* Correspondence may be addressed to: R. Fishel (rfishel@osu.edu) or J. Liu (liujiaquan@sibcb.ac.cn)

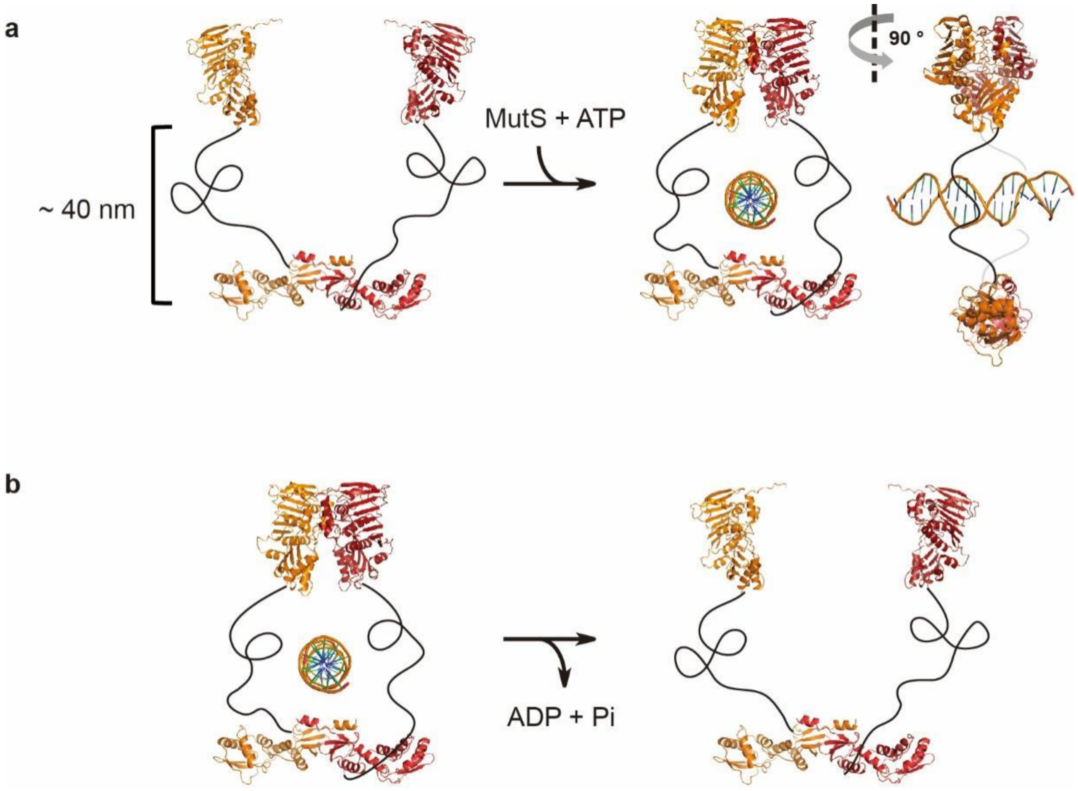

Supplementary Figure 1. Closing (top) and the opening (bottom) the MutL N-terminal domain. N- and C-terminus structure of MutL (PDB IDs: 1B63 or 1B62, and 1X9Z) joined by flexible linkers. (**a**) ATP binding-dependent dimerization of N-terminal domains forming a sliding clamp on the DNA. (**b**) ATP hydrolysis-dependent clamp opening and release from the DNA.

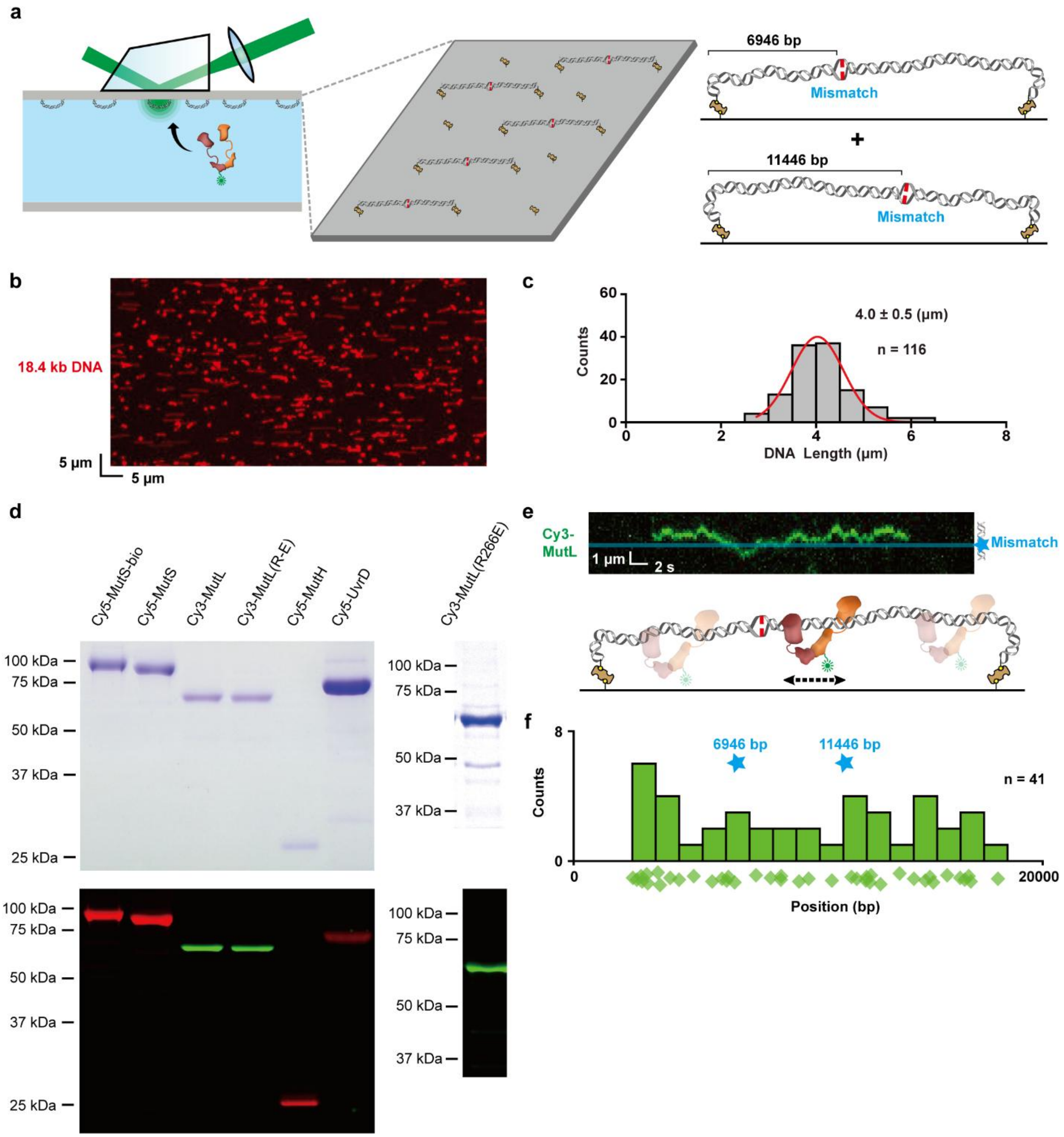

Supplementary Figure 2. Visualize MutL-DNA interactions by smTIRF. (**a**) An illustration of imaging MutL-DNA association using a single molecule total internal reflection fluorescence (smTIRF) system (left). Two possible orientations of the 18.4-kb mismatched DNA are shown (right). (**b**) Representative 18.4-kb mismatched DNA visualized by smTIRF microscopy in the absence of flow. The DNA was stained with Syto 59 and an 85 x 42.5 µm field of view is shown. (**c**) The length distribution of the mismatched DNA observed by smTIRF microscopy (n = number

of DNA molecules). The data was fit with a Gaussian distribution that determined the mean ± s.d. (**d**) Coomassie stained (top) and fluorescent (bottom) images of SDS-PAGE gels showing the labeled MMR proteins. (**e**) Representative kymograph and illustration showing the diffusion of a MutL-Cy3 along the mismatched DNA. 2 nM MutL-Cy3 protein in buffer containing 30 mM NaCl was injected into the flow cell. Blue star and line indicate the position of the mismatch. Dashed curved arrow indicates 1D diffusion. (**f**) The distribution of individual initial binding location for MutL bound to the DNA. Initial binding location of MutL is defined as the position of fluorophore in the first visible frame. Diamonds represent individual starting point and the blue stars indicate the two possible positions of the mismatch (n = number of events).

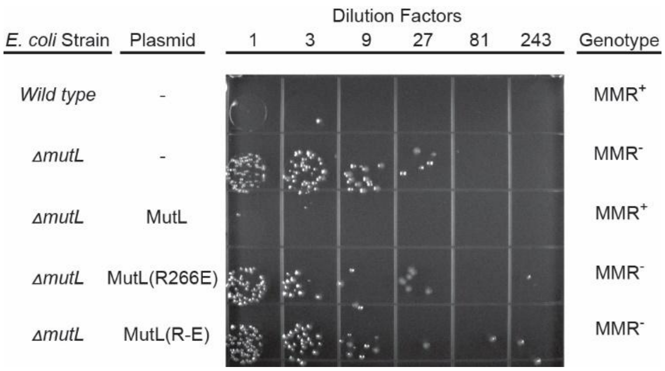

Supplementary Figure 3. Representative image of *in vivo* complementation assay. *Wild type* or *ΔmutL E.coli* cultures with or without complementing plasmids containing *wild type* MutL, MutL(R266E) or MutL(R-E) were diluted in L-broth and spotted onto LB plates containing 100 mg/ml rifampicin. The frequency of spontaneous rifampicin-resistant colonies is a semi-quantitative measure of mutation rate.

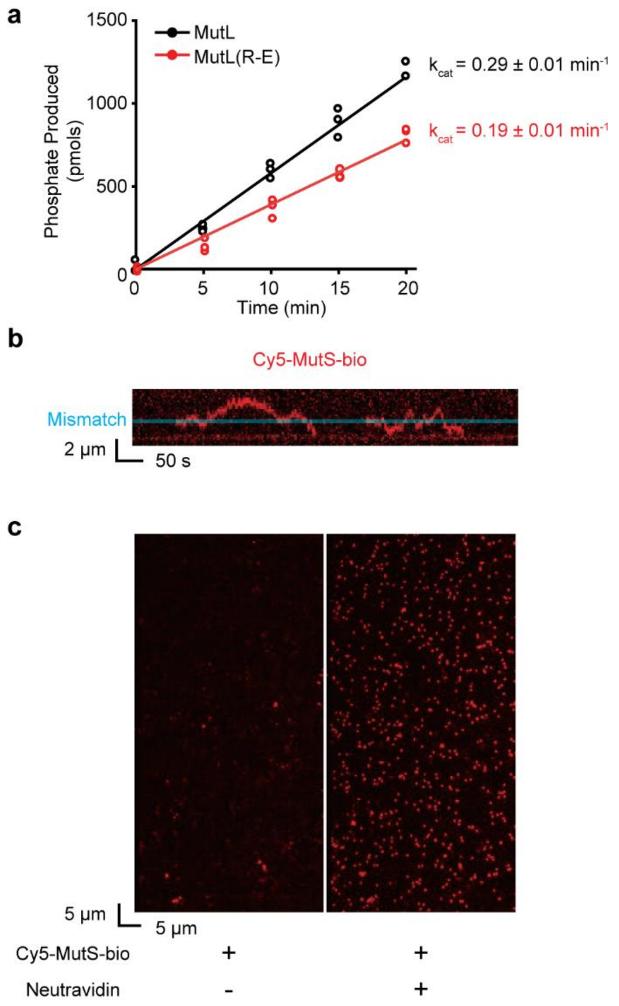

Supplementary Figure 4. ATPase activity of MutL, and Cy5-MutS-bio forms an ATP-bound sliding clamp on DNA. (**a**) ATP hydrolysis of MutL or MutL(R-E) measured at various time. Open circles represent individual numbers from at least two independent experiments. A linear function was fit to the data to derive the turnover number (k_{cat}) of MutL ATPase (mean ± s.e.). (**b**) Representative kymograph showing the diffusions of two individual Cy5-MutS-bio sliding clamps events on a single mismatched DNA (in the presence of neutravidin, see Methods). Blue line indicate the position of the mismatch. (**c**) Representative fluorescent image of neutravidin-immobilized MutS-bio-Cy5 molecules visualized by smTIRF microscopy. A 42 x 85 µm field of view is shown.

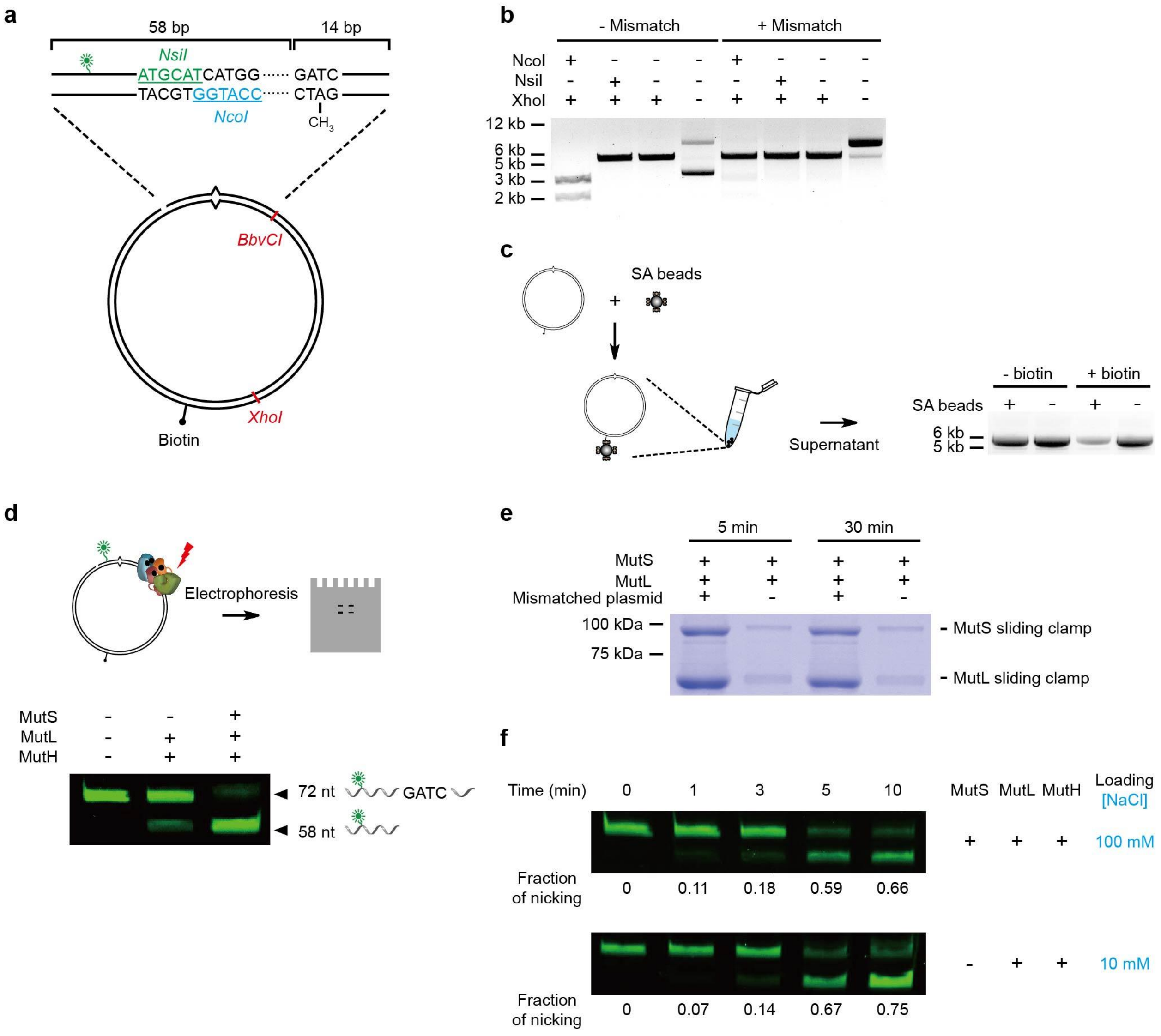

Supplementary Figure 5. MutH endonuclease analysis. (**a**) An illustration of the circular Cy3-biotin mismatched DNA used for MutH endonuclease analysis. This DNA substrate contains a single G/T mismatch between overlapping NsiI and NcoI restriction sites, a 5'-strand scission, a Cy3-label, a hemi-methylated GATC site and a biotin-label formed by annealing Oligo 7 and 8 between the four Nt.BbvCI sites within the modified pRSET-B plasmid (see: Methods; Supplemental Note 1). (**b**) Validation of the G/T mismatch. Non-mismatched and mismatched DNA were digested with NsiI or NcoI and XhoI and resolved on a 1% agarose gel. DNA containing a G/T mismatch is resistant to both NciI and NcoI. (**c**) Validation of biotin-label in the DNA substrate. Plasmid DNA was incubated with or without streptavidin coated superparamagnetic beads (SA beads), and the DNA in supernatant was collected and resolved on a 1% agarose gel. More than 60% of the biotin-DNA was immobilized by the SA beads (Lane 3). (**d**) Validation of the MutH endonuclease assay. Top: Illustration of MutH endonuclease analysis. Bottom: the circular Cy3-biotin mismatched DNA (Panel a) was first

incubated with MMR components detailed above under physiological ionic conditions (100 mM NaCl; see: Methods), digested by BbvCI to release the mismatch- and hemimethylated GATC-containing fragment and resolved on a 12% denaturing PAGE (see: Methods). MutH endonuclease cleavage at the hemimethylated GATC site resulted in a 58 nt product that was dependent on including MutS, MutL and MutH. (**e**) Precipitation of MutS and MutL sliding clamps with the circular Cy3-biotin mismatched DNA. MutS and MutL were first incubated with the circular Cy3-biotin mismatched DNA substrate (see **Fig. 6d**). The DNA was then precipitated using SA beads and washed with buffers to remove unbound proteins. The proteins bound to the washed and precipitated Cy3-biotin mismatched DNA substrate were released and analyzed on SDS-PAGE. (**f**) Kinetic analysis of MutH endonuclease by MutL sliding clamps loaded by either MutS or very low ionic strength conditions. MutL sliding clamps were first loaded in the presence of MutS under physiological ionic conditions (100 mM NaCl, top) or in the absence of MutS under very low ionic strength conditions (10 mM NaCl, bottom). The Cy3-biotin mismatched DNA substrate was washed and MutH endonuclease (100 nM, in buffer containing 100 mM NaCl) was then added and incubated for various times (see above). The reaction mix was then digested with BbvCI to release the mismatch- and hemimethylated GATC-containing fragment and resolved on a 12% denaturing PAGE (see: Methods). Densitometric quantification of the 58 nt band fraction compared to the total is shown below (fraction of nicking).

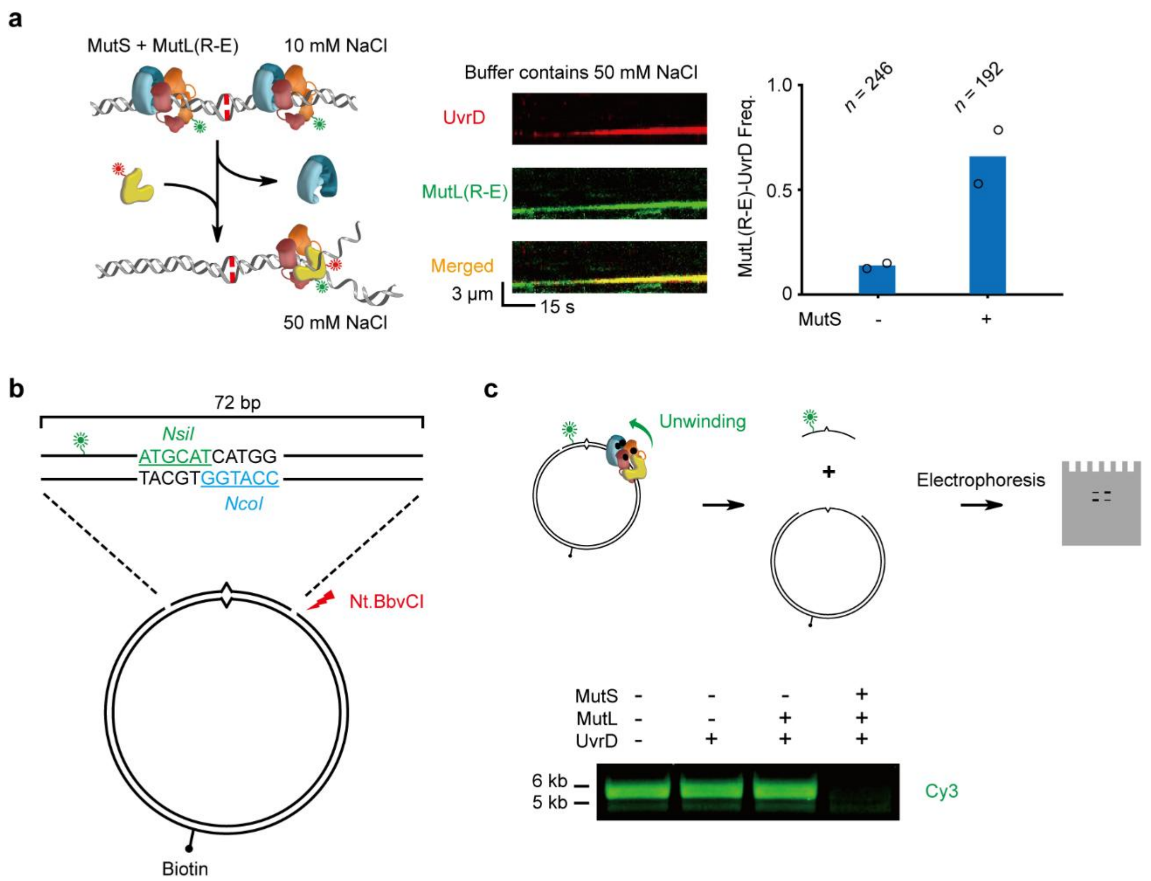

Supplementary Figure 6. UvrD helicase DNA unwinding analysis. (**a**) An illustration of the reaction sequence required to efficiently load MutL(R-E) sliding clamps and then examine UvrD helicase activity (left). MutL(R-E) sliding clamps were loaded at very low ionic conditions (10 mM NaCl), followed by buffer exchange to observe the interaction between a MutL(R-E) sliding clamp and UvrD (50 mM NaCl; see: Methods). Representative kymographs (middle) and frequency (right) of MutL(R-E) sliding clamps associated with UvrD helicase at low ionic strength (50 mM NaCl; n = total number of DNA molecules examined; columns represent mean values from two independent experiments). (**b**) An illustration of the Cy3-biotin mismatched DNA substrate containing a two adjacent strand breaks. The additional ssDNA break was introduced by Nt.BbvCI. (**c**) An illustration of the UvrD helicase DNA unwinding assay (top). Helicase unwinding releases the Cy3-labeled mismatch-containing strand from the circular Cy3-biotin mismatched DNA substrate. DNA substrate was incubated with MMR proteins at physiological ionic conditions (100 mM NaCl) and resolved on a 1% agarose gel. The decrease of Cy3 label requires MutS, MutL and UvrD (bottom).

Supplementary Table 1. Oligonucleotides

Name	Sequences
MutS for	TTCCCCTCTAGAAATAATTTTGTTTAACTTTAAGAAG
MutS rev1	GAAGAACCAGTTTCAGGTAAAGAGCCACCCCCTCCCACCAGGCTCTTCAA
MutS rev2	GAATTCGGATCCTTAATGATGATGATGATGATGAGAAGAACCAGTTTCAGGTAAAGA
MutS-bio for	TGGCTGCATATGCCAGTGCGCGATACCCGCGTGTTGCTT
MutS-bio rev1	ATGACCACCACCACCCACCAGGCTCTTCAAGCGA
MutS-bio rev2	ATATCATTCAACCCGGACCCATGATGATGATGATGATGACCACCACCACCC
MutS-bio rev3	CACCCCCTCCCTCATGCCATTCAATTTTCTGTGCTTCGAAAATATCATTCAACCCGGA
MutS-bio rev4	CGAATTCGGATCCTTAAGAAGAACCAGTTTCAGGTAAAGAGCCACCCCCTCCCTCATGC
MutL for	GATATACATATGCCAATTCAGGTCT
MutL rev1	ATGATGATGAGAAGAACCAGTTTCAGGTAAAGAACCACCACCCTCATCTTTCAGGGCT
MutL rev2	GTGGTGCTCGAGTTAATGATGATGATGATGATGAGAAGAACCAGT
MutH for1	CCTGAAACCGGAGGAGGATCTATGTCCCAACCTCGCCCA
MutH for2	GATATACATATGCATCACCATCACCATCATTCATCACTCCCTGAAACCGGAGGAGGA
MutH rev	CTGCTAGGATCCCTACTGGATCAGAAAATGACGG
UvrD for	TTCCCCTCTAGAAATAATTTTGTTTAACTT
UvrD rev1	TTCAGGTAAAGAGCCACCCCCTCCCACCGACTCCAGCCGGGC
UvrD rev2	GGCCGCAAGCTTTTAGTGATGGTGATGGTGATGAGAAGAACCAGTTTCAGGTAAAGAGC
Oligo 1	Phos-AGGTCGTCGCCCAGAAGATGGGCGAGTTTG
Oligo 2	Phos-GCCGCAAACTCGCCCATCTTCT
Oligo 3	Phos-TTCTTGGAGTCCACTGCAGTT/Biotin-TTCTGCAGTGGACTCCA
Oligo 4	Phos-CGCTTAGTGCTATGATGCGTT/Biotin-TTCGCATCATAGCACTA
Oligo 5	Biotin-CGCGGGTTTTCGCTATTTATGAAAATTTTCCGGTTTAAGGCGTTTCCGTTCTTCTTCGT
Oligo 6	Biotin-ACGAAGAAGAACGGAAACGCCTTAAACTGGAAAATTTTCATAAATAGCGAAAACCCGCG
Oligo 7	TCAGCATT/Cy3-AGGCTAGAGGTTAATGCATCATGGTATCAGATAGGTACATCGCATTACAG-GATCT GGAGCATCC
Oligo 8	Phos-TCAGCTCTCAGTATGCGAGCCGATCTTGAAGGCTTATTCGGTTAATTGCTAACTAT/Biotin-CC C

Supplementary Table 2. Labeling Efficiencies of *E.*coli MMR Proteins

Protein	Labeled monomer	Unlabeled Dimer	Dimer with a single fluorophore	Dimer with two fluorophores
MutS-Cy5	55%	20%	50%	30%
MutS-bio-Cy5	30%	49%	42%	9%
MutL-Cy3	46%	29%	50%	21%
MutL(R266E)-Cy3	57%	18%	50%	32%
MutL(R-E)-Cy3	35%	42%	46%	12%
MutH-Cy5	90%	N/A	N/A	N/A
UvrD-Cy5	10%	N/A	N/A	N/A

Supplementary Table 3. Diffusion Coefficients*

Protein configuration	[NaCl] mM	Mean ± Standard Deviation (10^{-3} μm^2 s^{-1})	Numbers of events
MutL	10	163 ± 77	30
	30	109 ± 89	33
	50	110 ± 50	32
MutS-MutL complex	100	5 ± 4	28
MutS-MutL(R-E) complex	10	4 ± 2	28
MutL sliding clamp	100	1628 ± 914	29
MutL(R-E) sliding clamp	100	2471 ± 1158	31

* Diffusion coefficients of different MutL configurations (data from **Fig. 1e, 2e and 4a**) are summarized.

Supplementary Table 4. Frequency of MutL Sliding Clamps*

MutS	MutL	ATP	[NaCl] mM	Frequency of MutL sliding clamps (Mean)
-	MutL	-	10	0.050
			30	0.015
			50	0.005
			100	0
-	MutL	+	10	0.250
			30	0.035
			50	0
			100	0
-	MutL(R266E)	+	10	0.063
			30	0
			50	0
			100	0
-	MutL(R-E)	+	10	0
			30	0
			50	0
			100	0
+	MutL	+	10	0.567
			30	0.467
			50	0.309
			100	0.073
+	MutL（R266E）	+	10	0.262
			30	0.226
			50	0.084
			100	0
+	MutL(R-E)	+	10	0.096
			30	0.055
			50	0.021
			100	0

* Frequencies of MutL sliding clamps (data from **Fig. 3d**) are summarized.

Supplementary Table 5. Frequency of MutL-MutH/UvrD Complexes*

Figures	MutS	MutL	MutH	UvrD	[NaCl] mM	Frequency of MutL-MutH/UvrD complexes (Mean)
**Fig. 6a**	-	MutL	+		100	ND
	+	MutL	+			0.289
	+	MutL(R-E)	+			ND
**Fig. 6c**	-	MutL(R-E)	+		10→100	ND
	+		+			0.132
**Fig. 7a**	-	MutL		+	100	ND
	+	MutL		+		0.234
	+	MutL(R-E)		+		0.008
**Fig. 7c**	-	MutL(R-E)		+	10→100	ND
	+			+		0.018

ND: not detected

* Frequencies of MutL-MutH/UvrD complexes (data from **Fig. 6a**, **6c**, **7a** and **7c**) are summarized.

Supplementary Note 1: Sequence of a modified pRSET-B plasmid

GATCTCGATCCCGCGAAATTAATACGACTCACTATAGGGAGACCACAACGGTTTCCCTCTAG

ACCTCAGCATTAGGCTAGAGGTTAATGCACCATGGTATCAGATAGGTACATCGCATTACAGGA

TCTGGAGCATCCTCAGCCATATGCCAATTCAGGTCTTACCGCCACAACTGGCGAACCAGATT

GCCGCAGGTGAGGTGGTCGAGCGACCTGCGTCGGTAGTCAAAGAACTAGTGGAAAACAGC

CTCGATGCAGGTGCGACGCGTATCGATATTGATATCGAACGCGGTGGGGCGAAACTTATCC

GCATTCGTGATAACGGCTGCGGTATCAAAAAAGATGAGCTGGCGCTGGCGCTGGCTCGTCA

TGCCACCAGTAAAATCGCCTCTCTGGACGATCTCGAAGCCATTATCAGCCTGGGCTTTCGC

GGTGAGGCGCTGGCGAGTATCAGTTCGGTTTCCCGCCTGACGCTCACTTCACGCACCGCA

GAACAGCAGGAAGCCTGGCAGGCCTATGCCGAAGGGCGCGATATGAACGTGACGGTAAAA

CCGGCGGCGCATCCTGTGGGGACGACGCTGGAGGTGCTGGATCTGTTCTACAACACCCC

GGCGCGGCGCAAATTCCTGCGCACCGAGAAAACCGAATTTAACCACATTGATGAGATCATC

CGCCGCATTGCGCTGGCGCGTTTCGACGTCACGATCAACCTGTCGCATAACGGTAAAATTG

TGCGTCAGTACCGCGCAGTGCCGGAAGGCGGGCAAAAAGAACGGCGCTTAGGCGCGATT

TGCGGCACCGCTTTTCTTGAACAAGCGCTGGCGATTGAATGGCAACACGGCGATCTCACG

CTACGCGGCTGGGTGGCCGATCCAAATCACACCACGCCCGCACTGGCAGAAATTCAGTATT

GCTACGTGAACGGTCGCATGATGCGCGATCGCCTGATCAATCACGCGATCCGCCAGGCCT

GCGAAGACAAACTGGGGGCCGATCAGCAACCGGCATTTGTGTTGTATCTGGAGATCGACC

CACATCAGGTGGACGTCAACGTGCACCCCGCCAAACACGAAGTGCGTTTCCATCAGTCGC

GTCTGGTGCATGATTTTATCTATCAGGGCGTGCTGAGCGTGCTACAACAGCAACTGGAAAC

GCCGCTACCGCTGGACGATGAACCCCAACCTGCACCGCGTTCCATTCCGGAAAACCGCGT

GGCGGCGGGGCGCAATCACTTTGCAGAACCGGCAGCTCGTGAGCCGGTAGCTCCGCGCT

ACACTCCTGCGCCAGCATCAGGCAGTCGTCCGGCTGCCCCCTGGCCGAATGCGCAGCCA

GGCTACCAGAAACAGCAAGGTGAAGTGTATCGCCAGCTTTTGCAAACGCCCGCGCCGATG

CAAAAATTAAAAGCGCCGGAACCGCAGGAACCTGCACTTGCGGCGAACAGTCAGAGTTTT

GGTCGGGTACTGACTATCGTCCATTCCGACTGTGCGTTGCTGGAGCGCGACGGCAACATTT

CACTTTTATCCTTGCCAGTGGCAGAACGTTGGCTGCGTCAGGCACAATTGACGCCGGGTGA

AGCGCCCGTTTGCGCCCAGCCGCTGCTGATTCCGTTGCGGCTAAAAGTTTCTGCCGAAGA

AAAATCGGCATTAGAAAAAGCGCAGTCTGCCCTGGCGGAATTGGGTATTGATTTCCAGTCAG

ATGCACAGCATGTGACCATCAGGGCAGTGCCTTTACCCTTACGCCAACAAAATTTACAAATC

TTGATTCCTGAACTGATAGGCTACCTGGCGAAGCAGTCCGTATTCGAACCTGGCAATATTGC

GCAGTGGATTGCACGAAATCTGATGAGCGAACATGCGCAGTGGTCAATGGCACAGGCCATA

ACCCTGCTGGCGGACGTGGAACGGTTATGTCCGCAACTTGTGAAAACGCCGCCGGGTGGT

CTGTTACAATCTGTTGATTTACATCCGGCGATAAAAGCCCTGAAAGATGAGGGTGGTGGTTC

TTTACCTGAAACTGGTTCTTCTCATCATCATCATCATCATTAACTCGAGCCTCAGCTCTCAGTA

TGCGAGCCGATCTTGAAGGCTTATTCGGTTAATTGCTAACTATCCCTCAGCAAGCTTGATCC

GGCTGCTAACAAAGCCCGAAAGGAAGCTGAGTTGGCTGCTGCCACCGCTGAGCAATAACT

AGCATAACCCCTTGGGGCCTCTAAACGGGTCTTGAGGGGTTTTTTGCTGAAAGGAGGAACT

ATATCCGGATCTGGCGTAATAGCGAAGAGGCCCGCACCGATCGCCCTTCCCAACAGTTGCG

CAGCCTGAATGGCGAATGGGACGCGCCCTGTAGCGGCGCATTAAGCGCGGCGGGTGTGG

TGGTTACGCGCAGCGTGACCGCTACACTTGCCAGCGCCCTAGCGCCCGCTCCTTTCGCTT

TCTTCCCTTCCTTTCTCGCCACGTTCGCCGGCTTTCCCCGTCAAGCTCTAAATCGGGGGCT

CCCTTTAGGGTTCCGATTTAGTGCTTTACGGCACCTCGACCCCAAAAAACTTGATTAGGGTG

ATGGTTCACGTAGTGGGCCATCGCCCTGATAGACGGTTTTTCGCCCTTTGACGTTGGAGTC

CACGTTCTTTAATAGTGGACTCTTGTTCCAAACTGGAACAACACTCAACCCTATCTCGGTCTA

TTCTTTTGATTTATAAGGGATTTTGCCGATTTCGGCCTATTGGTTAAAAAATGAGCTGATTTAA

CAAAAATTTAACGCGAATTTTAACAAAATATTAACGCTTACAATTTAGGTGGCACTTTTCGGGG

AAATGTGCGCGGAACCCCTATTTGTTTATTTTTCTAAATACATTCAAATATGTATCCGCTCATG

AGACAATAACCCTGATAAATGCTTCAATAATATTGAAAAAGGAAGAGTATGAGTATTCAACATT

TCCGTGTCGCCCTTATTCCCTTTTTTGCGGCATTTTGCCTTCCTGTTTTTGCTCACCCAGAA

ACGCTGGTGAAAGTAAAAGATGCTGAAGATCAGTTGGGTGCACGAGTGGGTTACATCGAAC

TGGATCTCAACAGCGGTAAGATCCTTGAGAGTTTTCGCCCCGAAGAACGTTTTCCAATGATG

AGCACTTTTAAAGTTCTGCTATGTGGCGCGGTATTATCCCGTATTGACGCCGGGCAAGAGCA

ACTCGGTCGCCGCATACACTATTCTCAGAATGACTTGGTTGAGTACTCACCAGTCACAGAAA

AGCATCTTACGGATGGCATGACAGTAAGAGAATTATGCAGTGCTGCCATAACCATGAGTGAT

AACACTGCGGCCAACTTACTTCTGACAACGATCGGAGGACCGAAGGAGCTAACCGCTTTTT

TGCACAACATGGGGGATCATGTAACTCGCCTTGATCGTTGGGAACCGGAGCTGAATGAAGC

CATACCAAACGACGAGCGTGACACCACGATGCCTGTAGCAATGGCAACAACGTTGCGCAAA

CTATTAACTGGCGAACTACTTACTCTAGCTTCCCGGCAACAATTAATAGACTGGATGGAGGC

GGATAAAGTTGCAGGACCACTTCTGCGCTCGGCCCTTCCGGCTGGCTGGTTTATTGCTGAT

AAATCTGGAGCCGGTGAGCGTGGGTCTCGCGGTATCATTGCAGCACTGGGGCCAGATGGT

AAGCCCTCCCGTATCGTAGTTATCTACACGACGGGGAGTCAGGCAACTATGGATGAACGAAA

TAGACAGATCGCTGAGATAGGTGCCTCACTGATTAAGCATTGGTAACTGTCAGACCAAGTTT

ACTCATATATACTTTAGATTGATTTAAAACTTCATTTTTAATTTAAAAGGATCTAGGTGAAGATC

CTTTTTGATAATCTCATGACCAAAATCCCTTAACGTGAGTTTTCGTTCCACTGAGCGTCAGAC

CCCGTAGAAAAGATCAAAGGATCTTCTTGAGATCCTTTTTTTCTGCGCGTAATCTGCTGCTT

GCAAACAAAAAAACCACCGCTACCAGCGGTGGTTTGTTTGCCGGATCAAGAGCTACCAACT

CTTTTTCCGAAGGTAACTGGCTTCAGCAGAGCGCAGATACCAAATACTGTTCTTCTAGTGTA

GCCGTAGTTAGGCCACCACTTCAAGAACTCTGTAGCACCGCCTACATACCTCGCTCTGCTAA

TCCTGTTACCAGTGGCTGCTGCCAGTGGCGATAAGTCGTGTCTTACCGGGTTGGACTCAAG

ACGATAGTTACCGGATAAGGCGCAGCGGTCGGGCTGAACGGGGGGTTCGTGCACACAGCC

CAGCTTGGAGCGAACGACCTACACCGAACTGAGATACCTACAGCGTGAGCTATGAGAAAGC

GCCACGCTTCCCGAAGGGAGAAAGGCGGACAGGTATCCGGTAAGCGGCAGGGTCGGAAC

AGGAGAGCGCACGAGGGAGCTTCCAGGGGGAAACGCCTGGTATCTTTATAGTCCTGTCGG

GTTTCGCCACCTCTGACTTGAGCGTCGATTTTTGTGATGCTCGTCAGGGGGGCGGAGCCTA

TGGAAAAACGCCAGCAACGCGGCCTTTTTACGGTTCCTGGCCTTTTGCTGGCCTTTTGCTC

ACATGTTCTTTCCTGCGTTATCCCCTGATTCTGTGGATAACCGTATTACCGCCTTTGAGTGAG

CTGATACCGCTCGCCGCAGCCGAACGACCGAGCGCAGCGAGTCAGTGAGCGAGGAAGCG

GAAGAGCGCCCAATACGCAAACCGCCTCTCCCCGCGCGTTGGCCGATTCATTAATGCAG

The plasmid contains four BbvCI/Nt.BbvCI recognition sites (colored in red) that were used for the introduction of a mismatch, a nick, a Cy3-labeled, a biotin-labeled and a hemi-methylated site.
